# Growth Performance, Body Measurements, Carcass and Some Internal Organs Characteristics of Pekin Ducks

**DOI:** 10.3390/ani9110963

**Published:** 2019-11-13

**Authors:** Dariusz Kokoszyński, Rafał Wasilewski, Mohamed Saleh, Dariusz Piwczyński, Henrieta Arpášová, Cyril Hrnčar, Martin Fik

**Affiliations:** 1 Department of Animal Sciences, Faculty of Animal Breeding and Biology, UTP University of Science and Technology, 85084 Bydgoszcz, Poland; rafwas9@wp.pl; 2 Department of Poultry and Animal Production, Faculty of Agriculture, Sohag University, 82524 Sohag, Egypt; bydg2016@gmail.com; 3 Department of Animal Biotechnology and Genetics, Faculty of Animal Science and Biology, UTP University of Science and Technology, 85084 Bydgoszcz, Poland; darekp@utp.edu.pl; 4 Department of Small Animal Science, Faculty of Agrobiology and Food Resources, Slovak University of Agriculture, 94976 Nitra, Slovak; henrieta.arpasova@uniag.sk (H.A.); Cyril.Hrncar@uniag.sk (C.H.); martin.fik@uniag.sk (M.F.)

**Keywords:** duck, conservation flock, growth, meat characteristics, intestine, correlations

## Abstract

**Simple Summary:**

The protection and conservation of native poultry breeds, including ducks, is important for biological, cultural, emotional and scientific reasons. The objective of this study was to compare three lines of Pekin ducks for growth performance, body measurements, carcass and digestive system characteristics. The results showed significant differences between the tested lines of ducks in body weight and body measurements, as well as a small number of significant differences in carcass and digestive system characteristics. This may suggest that the tested lines of ducks are different and distinct, mainly in terms of growth and body measurements.

**Abstract:**

Native breeds of ducks have been the subject of many studies in the past, yet the relevant knowledge is still incomplete and needs to be further expanded. The objective of this study was to provide information about differences in growth performance, dressing percentage, carcass composition and digestive morphometry among three lines of Pekin ducks from conservation flocks raised in Poland. The study used 180 sexed Pekin ducks—30 males and 30 females of line P33 (ducks of Polish origin), 30 males and 30 females of line P8 (ducks of Danish origin), and 30 males and 30 females of line P9 (ducks of French origin). Throughout the study (49 d), ducks were confined indoors in six pens. Birds were fed complete commercial diets ad libitum and had unrestricted access to water. The compared lines of ducks differed significantly in body weight from 1 to 49 d of age except of ducks of both sexes at 14 d. At 49 d of age, significant differences were observed between the tested ducks in all the body measurements. Duck genotype had a significant effect on preslaughter body weight, carcass weight and breast muscle, neck and remainders contents, caeca length, liver weight and gizzard percentage. The results show that the tested ducks were significantly different and unique, mainly in terms of the body biometric characteristics.

## 1. Introduction

The globalization and intensification of livestock production has marginalized or eliminated many native breeds and varieties of poultry [1]. According to the FAO (Food and Agriculture Organization of the United Nations) [2], 14.6% of birds and 17.8% of farmed mammals in Europe and the Caucasus, and 11.6% of birds and as much as 35.3% of farmed mammals in North America are critically endangered. Such a high proportion of critically endangered breeds of birds and farmed mammals on these continents is mainly related to the commercial large-scale production systems that eliminate or marginalize many native, less efficient breeds of poultry and farmed mammals [3]. In order to prevent the ongoing replacement and extinction of the native breeds of farm animals, special programs and conservation centers have been established in many countries of the world. In Poland, work to preserve the conservation flocks of ducks was started in the 1970s. Today, the genetic resources conservation program includes 10 conservation flocks of ducks: P11, P22, P33, P44 and P55 (Pekin of Polish origin, lines of ducks of different genotype, body size and productivity), P8 (Pekin of Danish origin), P9 (Pekin of French origin), LsA (Pekin of English origin), Mini Duck (Polish ducks bred from wild mallards—*Anas platyrhynchos* L. and Pekin ducks), and KhO1 (Polish hybrid of Khaki Campbell males and Orpington Fauve females) [4]. Ducks from conservation flocks, despite their poorer meat and reproductive performance, are more resistant to disease, adapt better to adverse and variable environmental and feeding conditions, and are more efficient in feed utilization. The meat of ducks from conservation flocks is fine-fibered and low in fat, while their eggs and feathers are of better quality than those from commercial duck hybrids. Conservation flocks are subjected to no selective breeding, they are only selected for conformation and health. Their management is dictated by biological, scientific, cultural, economic and emotional reasons [5].

In the past, populations of native duck breeds or lines were used to create new breeding strains. Pekin ducks of Danish origin (line P8) together with breeding ducks of line A44 were used to create a new breeding line A55. Currently, A55 birds are one of the two pedigree lines of Pekin ducks improved in Poland. For many years (until 2015), Polish Pekin ducks (of lines P44 and P55) were accepted for distribution as a stock for parental flocks of breeder ducks. In 2007, breeder flocks of P44 and P55 ducks accounted for as much as 94.2% of the Polish Pekin breeder duck farms [6], but in recent years, they were replaced by parental breeder flocks of English or French origin, which exhibit a higher level of reproductive traits [7].

Ducks from conservation flocks are highly suitable for game reserves, leisure parks and zoos as exhibition stock for amateur breeders, as well as being used for production of many local and national dishes. They have been the subject of much research in Poland [4,8,9,10] and abroad [11,12,13,14,15,16]. In recent years, only a few publications [4,17,18,19] have dealt with the growth traits and slaughter value of P33, P8 and P9 ducks, which are the subject of the present study. The earlier study showed significantly higher body weight of 56-day-old Pekin ducks of Danish origin (line P8) compared to Pekin ducks of French (line P9) and Polish origin (line P33). The carcasses of Pekin ducks of Danish origin had a significantly higher content of breast muscle and a higher abdominal fat percentage than those of Pekin ducks of French origin [4].

Results for the biometric characteristics of the digestive system of these duck lines at 49 d of age have not been presented to date. The objective of the study was to compare three lines of ducks: P8 (Pekin of Danish origin), P9 (Pekin of French origin) and P33 (Pekin of Polish origin) for body weight, body measurements, feed intake, feed conversion ratio, carcass and digestive system characteristics.

## 2. Materials and Methods

The duck experiment was conducted with the approval of the Local Ethics Committee for Experiments on Vertebrate Animals in Bydgoszcz, Poland (Resolution no.21 of 2014), as described in the approved application.

### 2.1. Birds and Housing

The study used 180 sexed Pekin ducks of lines P8 (Pekin of Danish origin), P9 (Pekin of French origin) and P33 (Pekin of Polish origin), which are included in the genetic resources conservation program in Poland. All the compared lines of Pekin ducks had white feathers, orange-yellow legs and beaks. The body weight of P33 and P8 old birds ranges from 3150 to 3400 g, and P9 old birds between 2850–3100 g [5].

Day-old sexed chicks (1:1 sex ratio) purchased from the National Research Institute of Animal Production in Kraków (Waterfowl Genetic Resources Station in Dworzyska), transported to the farm and assigned to groups according to line. Each line was divided into two subgroups, with 30 males and 30 females per subgroup. Throughout the 49 d growth period, ducks were kept in a conventional poultry building without outdoor access. Illumination was provided by incandescent lights. Up to 21 d, additional infrared heaters were used as a local source of heat and light. In the first week of age, temperature was 30–32 °C in the rearing area (under infrared heater) and 23–24 °C inside the building. It was later reduced by 2–3 °C each week under the heater and by 1 °C in the rearing area. From 22 d of age, air temperature was 21 ± 1 °C. Relative humidity during rearing was 60%–70%. Ducks were kept in six pens, each having an area of 12 m^2^. Each pen contained 30 males or 30 females of one line.

### 2.2. Feeding Program and Diets

From 1 to 21 d of age, birds were fed a complete commercial diet for fattening ducks in crumble form. The starter diets contained 20.7% CP (crude protein) and 12.5 MJ (2996 kcal) ME (metabolizable energy) per kilogram feed. From 22 to 49 d of growth, ducks received a complete commercial grower/finisher diet (in granulate form) containing 17.5% CP and 12.5 MJ (2985 kcal) ME. The ingredient composition of the starter and grower/finisher diets fed to the ducks during the study is presented in Table 1. Information about the basic chemical composition, energy value and content of some amino acids in both diets is shown in Table 2. Throughout the study (49 d), birds were fed complete commercial diets ad libitum and had unrestricted access to water.

### 2.3. Growth Performance

During the 49 d growth period, body weight, feed intake and mortality were recorded. Ducks were individually weighed at 1 d of age with an electronic balance (Radwag PS 1000.R2, Radwag, Radom, Poland) to within 0.01 g and at 7, 14, 21, 28, 35, 42 and 49 d of age with an electronic hook scale (Axis BD15S, Axis, Gdańsk, Poland) to within 5 g. During the whole rearing period, the weight of complete diets was recorded daily and the amount of feed refusals at the end of each week of rearing. This enabled the calculation of the average daily intake of feed per bird (separately for males and females) and its conversion (g feed/g gain) using the feed conversion ratio (FCR). No mortality occurred during the experiment.

### 2.4. Body Measurements

After the determination of body weight at 49 d of age, ducks were tape-measured with an accuracy of 1 mm for length of trunk with neck (between the first cervical vertebra and posterior superior tuberosity of the ischium), trunk length (between the tuberosity of the shoulder joint and posterior tuberosity of the ischium), keel length (from the anterior to the posterior edge of the keel), chest circumference (behind wings through the anterior edge of the keel and middle thoracic vertebra), breast muscle thickness (on the right carcass side using a needle probe, 1.5 cm off the keel and 4 cm from its start), drumstick length (from the knee joint to the hock joint), and shank length (from the hock joint to the lower surface of digit IV at its base).

### 2.5. Carcass Analysis

At 49 d of age, seven males and seven females of each line were selected for carcass dissection and evaluation of digestive tract morphometry. Birds whose body weight was closest to the arithmetic mean of male or female body weight in a given line were selected for slaughter. A total of 42 birds were selected for slaughter. The mean weight of the ducks selected for dissection was 2007 ± 70.5 g (line P33), 2153 ± 133.2 g (line P8), 1979 ± 124.9 g (line P9), 2055 ± 107.9 g (males), and 2037 ± 159.7 g (females). Ten hours before slaughter, ducks were subjected to feed but not water withdrawal. Slaughter, scalding, defeathering and evisceration were performed on the farm. Water temperature was about 65 °C and scalding time was about 1.5 min. Birds were stunned with a club and the blood vessels in the neck were cut for bleeding. Eviscerated carcasses with neck were chilled in a Hendi chill cabinet (Hendi, Gądki, Poland) at 4 °C for 18 h. After removal from the cabinet, the chilled carcasses were individually weighed on a WLC 6/12/F1/R electronic balance (Radwag) to within 0.1 g. Next, whole eviscerated carcasses with neck (without head) were dissected in accordance with the method developed and described by Ziołecki and Doruchowski [20]. Each carcass was dissected into abdominal fat, neck without skin, wings with skin, skin with subcutaneous fat from the whole carcass without wings skin, breast muscles (major and minor pectoral muscles), leg muscles (all thigh and drumstick muscles), and remainder of the carcass (skeleton with some skeletal muscles). The dissected carcass parts were weighed on the above balance to the nearest 0.1 g, and their proportion (%) in eviscerated carcass with neck (without giblets) was calculated.

### 2.6. Digestive System Characteristics

After evisceration, prior to carcass chilling, the heart, gizzard (without digesta), liver (without gallbladder) and spleen were separated from each duck. The internal organs were weighed on a Radwag PS 1000.R2 electronic balance (Radwag) to the nearest 0.1 g and their percentage in body weight of the ducks selected for slaughter was calculated. The intestines were also separated during evisceration. A dressmaker’s tape was used to determine (±1 mm) small intestine segment (duodenum, jejunum, ileum), caeca and colon lengths, after which the total intestinal length was calculated. The length of duodenum was measured from the pylorus to pancreatic loop, the length of jejunum from the pancreatic loop to Meckel’s diverticulum, and the length of ileum from Meckel’s diverticulum to the ileocaecal junction. The length of the colon was measured as the distance from the mouth of the caeca to the cloaca. The length of caeca was measured from the mouth of the ileum to vertex. The diameter of individual segments (the anterior, middle and posterior parts of the intestine) was measured with electronic calipers to the nearest 0.01 mm.

### 2.7. Statistical Analysis

The numerical data obtained for body weight, body measurements, feed intake, feed conversion ratio, dressing percentage, weight and percentage of carcass and some internal organs, as well as the length and diameter of intestinal segments of the three lines of ducks from genetic resources flocks, were subjected to statistical analysis. In the first stage of the analysis, we used the Shapiro-Wilk test to determine if empirical distribution of the traits was the same as the normal distribution. Arithmetic means as well as standard error of the mean (collectively for all lines) were calculated for each tested trait. The effect of line and sex on the analyzed duck traits was determined. The following linear model was used: *Y_ijk_* = *μ* + *a_i_* + *b_j_* + (*a* · *b*)*_ij_* + *e_ijk_* where *Y_ijk_*—value of the analyzed trait, *μ*—overall mean for the tested trait, *a_i_*—effect of *i*-th line of the genetic resources ducks, *b_j_*—effect of *j*-th sex, (*a* · *b*)*_ij_*—line by sex interaction, *e_ijk_*—random error.

In the next stage of the statistical analysis, the calculated arithmetic means were used to produce growth curves for the duck lines and sexes, which are presented in the figures. Due to their practically linear course, changes in body weight in successive test periods were modeled using first degree linear function. The use of this linear function for modeling the body weight was based on the calculated coefficients of determination, the values of which were close to 0.99.

Pearson’s coefficients of correlation were estimated between body weight and measurements, carcass traits (carcass weight, dressing percentage, carcass components) and digestive system characteristics (length and diameter of intestinal segments, weight and percentage of some organs).

Statistical calculations were made using the SAS package, version 9.4 (SAS Institute Inc., Gary, NC, USA) [21]. Significant differences between the mean values of the traits of the ducks from the compared lines as well as between males and females were determined using Tukey’s test. Differences were considered significant at *p* < 0.05.

## 3. Results

### 3.1. Growth Performance

The analysis of mean body weights of the drakes and ducks of different lines from 1 to 49 d of age showed significant (*p* < 0.05) differences in this trait except for 14 d of age (Table 3). The body weight of day-old P8 and P9 ducks of both sexes was significantly (*p* ≤ 0.05) higher than that of P33 birds. P33 and P9 ducks had a significantly greater body weight at 7 d of growth compared to P8 birds. At 14 d, ducks of the compared lines had similar (*p* > 0.05) body weights. At 21 d, P8 birds were the heaviest and P9 birds were the lightest with a significant difference (*p* < 0.05). From 28 to 49 d of age, P8 ducks of both sexes had a significantly (*p* < 0.05) greater body weight compared to P9 and P33 birds. The results also point to significant differences between males and females in body weight at 1, 7 and 21 d of age. The genotype by sex interaction was significant (*p* < 0.001) except for 1 d of age.

**Figure 1:**
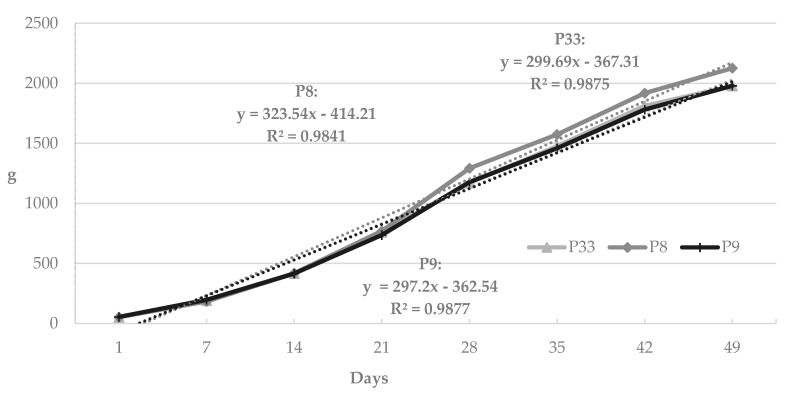
Curves for the body weight of Pekin ducks from three conservation flocks during rearing.

**Figure 2:**
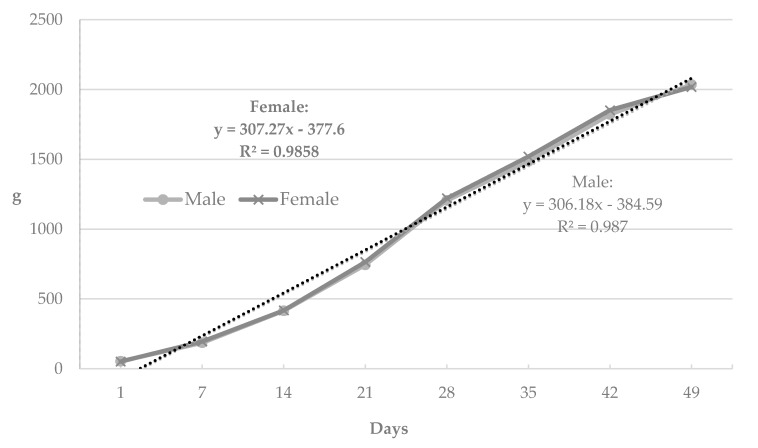
Curves for the body weight of males and female Pekin ducks from conservation flocks during rearing.

Figure 1 presents the curves illustrating body weight changes in the compared lines of the ducks. The shape of the curves was close to linear, and weekly body weight gain ranged from 297.20 to 323.54 g, depending on the line. It is also worth noting that up to 21 days of age, the birds of the studied lines showed a very similar growth. Starting from 3 weeks of age, P33 ducks exhibited a more dynamic body weight increase compared to P8 and P9 ducks—the dynamics of growth in these lines was highly similar. The curves in Figure 2 show practically the identical dynamics of changes in the body weight of drakes and ducks from 1 to 49 days of age, with the weekly body weight gain in the compared sex groups ranging from 306.18 (males) to 307.27 g (females).

In the present study, changes in the body weights of the ducks in successive test periods were modeled using the first degree linear function and a very high fit of the model was obtained. The very high coefficients of determination (from 0.9841 to 0.9877) are indicative of the very good predictive ability of the linear model based on the first degree function.

The data given in Table 4 suggest that at 49 d of age, duck genotype had a significant (*p* < 0.05) effect on all the body measurements studied. Pekin ducks of Danish origin (line P8) had significantly greater trunk length with neck, keel, drumstick and shank lengths as well as greater chest circumference compared to P9 and P33 ducks. Trunk length was greater in 49-d-old P8 and P33 ducks than in P9 birds. Breast muscle thickness was significantly smaller in P33 compared to P8 and P9 ducks. Regardless of the genetic origin, at 49 d, males exhibited significantly greater trunk length with neck, trunk, drumstick and shank lengths, and smaller keel length and breast muscle thickness compared to females.

No significant differences were noted between the males and females in chest circumference at 49 d (*p* = 0.660). The genotype by sex interaction for the body measurements of 49-d-old ducks was significant (*p* < 0.05) except for thickness of breast muscles and shank length.

The lowest intake of starter diet per day per bird, which was fed up to 21 d of age, was observed in P33 ducks from 1 to 14 d and in P9 ducks from 15 to 21 d. The highest average daily feed intake was noted in P8 (1 and 3 wk) and P9 bids (2 wk). The average daily intake of grower/finisher diet, which was fed from 22 to 49 d, was highest in P8 birds and lowest in P33 (22–42 d) or P9 ducks (43–49 d). Regardless of the genetic origin, average daily feed intake was lower in males than in females except for 5 wk (29–35 d) and 7 wk (43–49 d) of growth. During the entire experimental period (1–49 d) feed intake (g/bird/d) was higher in males than in females (Table 5).

The compared lines of the ducks from genetic resources flocks were characterized by poor feed conversion, especially between 43 and 49 d of age (Table 5). Feed conversion ratio (FCR) was best for P9 ducks from 1 to 7 d, for P8 ducks from 8 to 28 d, for P33 ducks from 29 to 42 d, and for P9 ducks from 43 to 49 d. During the whole study (1 to 49 d), P33 birds had the best FCR and P9 birds the worst FCR. Regardless of the genetic origin, males had better FCR at 2, 4, 6 and 7 wk than females, and females exhibited better FCR than males at 1, 3 and 5 wk (Table 5).

### 3.2. Carcass Charact Eristics

The average body and carcass weights of P8 ducks selected for slaughter at 49 d of growth was significantly (*p* < 0.001) higher than that of P9 and P33 birds (Table 6). Furthermore, genotype and sex as well as the genotype by sex interaction had a significant effect on the weight of eviscerated carcass with neck. The compared lines of ducks exhibited a similar dressing percentage at 49 d of age. The effects of genotype and sex as well as the genotype by sex interaction were not significant. Duck origin had a significant (*p* < 0.05) effect on the percentage of breast meat, neck, and carcass remainders. Males showed significantly (*p* < 0.05) higher carcass weight and neck percentage of carcass compared to females. For the other carcass traits, no significant differences were found between males and females. The genotype by sex interaction was not significant for the content of different carcass parts (Table 6).

### 3.3. Digestive System Characteristics

The tested lines of the ducks did not differ significantly (*p* > 0.05) in the length of small intestine segments (duodenum, jejunum, ileum), colon length and total intestine length. P8 and P33 ducks. Significantly longer caeca were found in males than in females. The genotype by sex interaction for the length of intestine and its segments was not statistically significant (Table 7).

The tested ducks did not differ in the diameter of different intestinal segments. The effects of sex as well as the genotype by sex interaction were not significant also for the diameter of different small intestine segments (duodenum, jejunum, ileum), and the lengths of caeca and colon. P9 ducks aged 49 d had significantly (*p* < 0.05) heavier liver compared to P33 and P8 birds. In turn, P8 ducks had non-significantly heavier heart, gizzard and spleen (*p* > 0.05) in comparison to P9 and P33 birds. Males had heavier gizzard, liver and spleen, and lighter heart compared to females.

Genotype also had a significant effect on gizzard percentage, and sex on liver percentage in the body weight of ducks at 49 d of age. P8 and P33 ducks had significantly higher gizzard proportion compared to P9 birds, and males compared to females showed a significantly higher liver percentage in body weight at 49 d of age. The genotype by sex interactions for the weight and percentage in the body weight of main internal organs in 49-d-old Pekin P8, P9 and P33 ducks were not significant (Table 8).

### 3.4. Correlations between Traits

Most morphometric body measurements were significantly and positively correlated with body weight. Carcass weight was significantly and positively correlated with keel length and breast muscle thickness, dressing percentage significantly and positively correlated with chest circumference, and percentage carcass remainders were significantly and negatively correlated with trunk length.

According to the scale of Stanisz [22], high correlation coefficients (r = 0.5–0.7) were only estimated between body weight and chest circumference and between body weight and keel length (Table 9).

The results show significant and positive correlations between body weight and gizzard percentage, and between trunk length with neck and length of caeca and heart weight. Positive and significant correlations were determined between keel length and jejunal diameter, negative coefficients of correlation between drumstick length and gizzard weight and percentage, and positive and significant coefficients of correlations between shank length and caeca length and diameter (Table 10 and Table 11).

The analysis of the correlation coefficients between carcass traits and digestive system characteristics showed significant and positive correlations between breast muscle content and spleen weight and percentage in the body as well as positive coefficients of correlation between the content of skin with subcutaneous fat and colon length. Significant and negative values of Pearson’s correlations were estimated between carcass neck percentage and ileum length (Table 12 and Table 13).

## 4. Discussion

The results of this study provide information about the body weight, body measurements, feed conversion ratio, slaughter value, carcass composition and digestive tract morphometry of the P8, P9 and P33 ducks that are included in the conservation program in Poland. An earlier study [18] demonstrated a slightly lower body weight of P8, P9 and P33 ducks up to 28 d of age compared to our findings. The average body weight of the P8, P9 and P33 ducks tested at 49 d was similar to the average body weight of 49-d-old ducks of the same origin in earlier studies [23,24], which may suggest that the conservation program for the conservation flocks of the ducks is highly effective in terms of their body weight. In our study, males had lower body weight than females until 6 wk of age, which is reflected in the results of an earlier study [18] with ducks from conservation flocks.

In the study by Witkiewicz et al. [25], the average body weight of 49-d-old P33 ducks of both sexes was 2860 g, which is more than in the ducks of the same line in our study. The differences between ducks of the same origin in body weight may be indicative of a significant effect of diet and environment on the value of this trait. The compared duck lines had the highest weight gains between 22 and 28 d of age. In an earlier study [18], P9 and P33 ducks showed the highest weight gains between 22 and 28 d, and P8 birds between 29 and 35 d. Kim et al. [26], who studied native lines of meat-type Korean ducks, observed the highest body weight gains between 15 and 28 d of age. Heo et al. [27] found the highest weight gains in commercial hybrids of Korean native ducks to also occur between 15 and 28 d. The same experiment [27] found markedly slower weight gains of ducks after 42 d of growth, which is consistent with our findings. The evaluated duck lines showed significant differences in all the body measurements taken on d 49, which may suggest that they are distinct and unique in terms of body biometric characteristics. Kisiel [18] found greater chest circumference and keel length in P8, P9 and P33 ducks aged 49 d compared to the ducks of the same origin and age tested in our study. In a later study [19], keel length in 49-d-old P8, P9 and P33 ducks was similar to our findings. In turn, Mazurowski et al. [28] found greater trunk length, keel length, shank length, and smaller trunk length with neck in 49-d-old commercial hybrids of Pekin ducks (AF51) in comparison with the Pekin ducks of lines P8, P9 and P33 evaluated in our study. However, compared to the tested P8, P9 and P33 ducks, AF51 hybrids showed a higher body weight at 49 d of age. Regardless of genetic origin, in our study 7-wk-old males had smaller chest circumference, smaller breast muscle thickness, and longer shanks, which is reflected in the findings of a study [29] conducted with P45 and P54 hybrid ducks resulting from crosses with P44 and P55 ducks from conservation flocks.

The evaluated lines of ducks were characterized by a poor feed conversion ratio. From 1 to 49 d of age, FCR ranged from 3.14 kg (line P33) to 3.25 kg (line P9). In the study by Kisiel [18], FCR was 2.84 kg in P8 ducks and 2.92 kg in P33 ducks, and the highest value (3.16 kg) was obtained for P9 ducks. In the study by Heo et al. [27] with Korean native ducks, FCR ranged from 3.01 to 3.26 kg between 1 and 56 d of age. Heo et al. [27] also found FCR to drastically increase at the end of the rearing period. Between 43 and 56 d of age, Korean native ducks consumed from 8.0 to 10.6 kg feed per kg gain. In our study, we also observed a significant increase in FCR during the final week of growth, which was probably due to the significant decrease in body weight gains in this period.

At the age of 49 d, the compared lines of ducks from conservation flocks showed lower dressing percentage compared to those (62.1%–65.0%) reported by Książkiewicz [30]. In the study by Górski [31], P11, P22, P44 and P55 ducks from genetic resources flocks exhibited similar or lower dressing percentage (from 55.9% to 59.2%) at 49 d of age. In our study, the breast meat yield in the carcasses of 49-d-old ducks of lines P8, P9 and P33 was lower than in ducks of the same genotype and age investigated by Książkiewicz [30]—breast meat yield for eviscerated carcasses with neck was 11.0% (line P9), 11.2% (line P33) and 12.7% (line P8). Another experiment [18] found lower breast meat yield in the carcasses of 49-d-old P8 (10.2%), P9 (8.6%) and P33 ducks (9.2%). In 56-d-old P8, P9 and P33 ducks, Gornowicz and Szukalski [32] found higher breast meat yield (15.2%, 12.5% and 14.3%, respectively) in comparison to 49-d-old ducks of the same origin, which may suggest that the 56-d-old ducks of the studied lines are more suitable for the consumers. In 49-d-old P11 and P22 genetic resources ducks, Kokoszynski and Bernacki [33] also observed low breast meat yield (P11—10.8%, P22—10.7% of the carcass). The results of studies with commercial hybrids of Pekin ducks [34,35] show better development of breast muscle in the ducks improved for this trait. Leg meat yield in P8, P9 and P33 ducks was lower than that reported by Kisiel [18] and Książkiewicz and Kiełczewski [36]. The content of skin with subcutaneous fat in eviscerated carcasses from 49-d-old ducks of the tested lines exceeded 27% and was higher than in 49-d-old ducks of the same genotype evaluated by Książkiewcz and Kiełczewski [36]. In the study by Kokoszyński and Bernacki [33], the proportion of skin with subcutaneous fat in 49-d-old ducks from conservation flocks was 30.0% in line P11 and 30.3% in line P22. These results confirm the unfavourably high fatness of the carcasses from genetic conservation ducks that are not selected for rapid growth rate (which shortens the rearing period) and for less fatty carcasses. A lower percentage of skin with subcutaneous fat in carcasses was observed in commercial hybrids of Pekin ducks [35]. The abdominal fat content in the carcasses of 49-d-old ducks of the compared lines was high (1.4%–1.6% of the carcass). A lower abdominal fat content in the carcasses of Pekin ducks was reported by Kwon et al. [14] and Kokoszyński et al. [35], among others. The carcasses of P8, P9 and P33 ducks had a high remainders content. An earlier study [35] found lower carcass reminders in commercial hybrids of Pekin ducks.

In our study, we also determined the length and diameter of individual intestinal segments of the P8, P9 and P33 ducks aged 49 d. An earlier study [37] with 49-d-old commercial hybrids of Pekin ducks (SM3 Heavy or AF51) found longer jejunum, ileum and caeca, and shorter duodenum and colon in comparison to our results. Furthermore, this experiment [36] did not confirm a significant effect of sex on caeca length observed in our study. Kokoszyński et al. [38], who evaluated P9 Pekin ducks after two reproductive seasons, observed that individual intestine segments were longer except for duodenum when compared to P9 ducks evaluated at 49 d. It was also found that the compared lines of P8, P9 and P33 ducks had lower diameters of duodenum, jejunum, ileum, caeca and colon when compared with the diameters of intestinal segments in commercial hybrids of Pekin ducks (SM3 Heavy or AF51) [37]. The differences in intestinal length and diameter have an effect on the intestinal villus area and nutrient absorption, which influences productive traits in poultry. The intestinal length and diameter are considerably influenced by the amount of feed intake, body size, species, breed, age, gender, health, and physiological status of the birds [39].

Pekin ducks of the tested lines had medium liver, gizzard, heart and spleen weights. In slightly heavier Pekin drakes (2559 g) and ducks (2413 g) aged 49 d, Bons et al. [40] noted generally higher weights of liver (44.3 g), gizzard (74.5 g) and heart (14.5 g) in comparison with our study. The same evaluation found higher liver, heart and gizzard percentages in the bodies of 49-d-old Pekin ducks when compared to the conservation ducks in our study. Wasilewski et al. [37], who evaluated digestive morphometry of 49-d-old commercial hybrids of Pekin ducks, observed lower gizzard, liver and heart percentage, and higher spleen percentage compared to the P8, P9 and P33 ducks of the same age. In our study, males, compared to females, had higher gizzard and lower heart percentage, which is consistent with the findings of Stęczny et al. [41] obtained for commercial hybrids of Pekin ducks.

This study also estimated the coefficients of correlation between body weight and dimensions, and carcass traits. In the study by Kokoszyński [29], the correlation coefficients between the weight of eviscerated carcass with neck and the body weight and dimensions in commercial hybrids of Pekin ducks aged 6–7 wk were positive, except for the correlation with shank length, and the estimated Pearson’s correlations were higher than in our study. In the same experiment [29], dressing percentage showed positive and low correlations with body weight, breastbone length, and chest circumference, which is consistent with our results. Furthermore, Kokoszyński [29] found that dressing percentage correlated negatively with trunk with neck length, trunk length, and shank length. Another experiment [42] estimated negative correlations between dressing percentage and body dimensions. One exception was the correlation between dressing percentage and breast muscle thickness, which assumed positive values. The present results show that chest circumference and drumstick length could be a good indicator of breast and leg muscle percentages in eviscerated carcasses of the studied duck lines. In the experiment by Kokoszyński [29], the best indicators of breast muscle percentage were body weight, chest circumference and shank length, while leg muscle percentage was best reflected in body weight (positive correlation) and in chest circumference and breast muscle thickness (negative correlation). Another study [43] demonstrated that the best indicator of breast muscle percentage is keel length and breast muscle thickness, while shoulder length, chest width and shank length are the best indicators of leg muscle percentage. In the study by Kokoszyński [29], percentage of skin with subcutaneous fat correlated negatively with shank length, and positively with trunk with neck length, breastbone length, chest circumference and breast muscle thickness, which was partly confirmed in our study. Yet another experiment [44] estimated negative correlations between breast muscle thickness and percentage of skin with subcutaneous fat, but this was not confirmed by our results.

## 5. Conclusions

Our results show the effectiveness of the conservation program for the conservation flocks of the ducks, as reflected in the slight changes in body weight, body dimensions, and carcass components in comparison with the results of studies performed more than a dozen years ago. The evaluated duck lines differed mainly in body weight during the growth period and in body measurements on day 49, suggesting that they are distinct and unique, which is essential for preserving biodiversity in the duck populations. The present study provided information about the biometric characteristics of the digestive system in young Pekin ducks of different origin. In the future, this research should be extended to include evaluation and comparison of the traits growth performance, carcass composition and the digestive system of adult ducks.

## Figures and Tables

**Table 1:** Ingredients of the diets for ducks.

Ingredients (% of Feed)	Starter	Grower/Finisher
	1−21 d	22−49 d
Corn	33.42	43.79
Wheat	25.00	25.00
Ground wheat	2.00	2.50
Soybean meal (465 g CP/kg)	27.50	17.90
Sunflower seed meal (390 g CP/kg)	1.50	2.00
Rape seed meal (347 g CP/kg)	1.00	1.00
Corn DDGS (280 g CP/kg)	3.00	3.00
Soybean oil	3.30	2.00
Limestone	1.29	0.99
Monocalcium phosphate	1.06	0.74
Sodium chloride	0.272	0.237
Sodium bicarbonate	0.10	0.14
DL-methionine	0.05	0.105
L-Lysine	0.008	0.098
Vitamin-mineral premix ^A^	0.50	0.50

^A^ 1 kg of vitamin-mineral premix provided: retinol 10,000 IU, cholecalciferol 2500 IU, α-tocopherol 20.00 mg, thiamine 0.5 mg, riboflavin 5.00 mg, niacinamide 20.00 mg, pyridoxine 1.0 mg, cobalamine 0.02 mg, folic acid 0.5 mg, menadione 2.5 mg, choline chloride 200.00 mg, Fe 45.00 mg, Mn 62.5 mg, Zn 50.00 mg, Cu 5.00 mg, Se 0.25 mg, I 1.3 mg.

**Table 2:** Chemical composition of the diets for ducks.

Ingredients (% of Feed)	Starter	Grower/Finisher
	1−21 d	22−49 d
DM	90.8	89.2
CP	20.7	17.5
Crude fat	5.7	5.0
Crude fiber	4.5	3.5
Crude ash	4.9	4.3
N–free extracts	55.0	58.9
ME ^A^ (kcal/kg of fed)	2996	2985
Calculated composition (%)		
Lysine	1.13	0.89
Methionine	0.46	0.41
Threonine	0.80	0.62
Tryptophan	0.26	0.21
Calcium	0.91	0.82
Phosphorus soluble	0.65	0.52

^A^ The values are calculated from ingredient AME values.

**Table 3:** Body weight (g) of Pekin ducks from conservation flocks during rearing.

Time	Genotype			Sex		SEM	*p* Value		
	Polish Pekin P33 (*n* = 60)	Danish Pekin P8 (*n* = 60)	French Pekin P9 (*n* = 60)	Male (*n* = 90)	Female (*n* = 90)		Genotype (G)	Sex (S)	G × S
1 d	51.3 b	52.8 a *	54.0 a	53.6	51.8 *	0.5	0.001	0.003	0.721
7 d	191 b	183 c *	197 a	186	195 *	2.3	0.001	0.002	0.001
14 d	415	418	416	415	418	4.6	0.851	0.509	0.001
21 d	759 ac*	770 a *	736 bc*	744	765 *	7.9	0.001	0.014	0.001
28 d	1170 b	1292 a	1177 b	1205	1221	14.7	0.001	0.250	0.001
35 d	1476 b	1574 a	1459 b	1486	1520	18.2	0.001	0.054	0.001
42 d	1811 b	1918 a	1781 b	1821	1852	22.5	0.001	0.174	0.001
49 d	1977 b	2126 a	1979 b	2035	2018	26.6	0.001	0.248	0.001

a–c—mean values of traits in rows, marked with different letters, differ significantly between lines (*p* < 0.05). *—statistically significant differences determined between males and females.

**Table 4:** Body dimensions of 49-d-old Pekin ducks from conservation flocks.

Dimension	Genotype			Sex		SEM	*p* Value		
	Polish Pekin P33 (*n* = 60)	Danish Pekin P8 (*n* = 60)	French Pekin P9 (*n* = 60)	Male (*n* = 90)	Female (*n* = 90)		Genotype (G)	Sex (S)	G × S
TN, cm	43.0 b*	43.8 a	42.4 c *	43.9	42.3 *	0.2	0.001	0.001	0.001
TL, cm	25.6 a*	25.6 a	24.3 b *	25.6	24.8 *	0.2	0.001	0.001	0.001
CC, cm	29.6 b	30.2 a	28.7 c	29.4	29.5	0.2	0.001	0.660	0.001
KL, cm	11.1 b	11.4 a *	10.8 c *	10.9	11.1 *	0.1	0.007	0.001	0.001
TB, cm	1.29 b	1.32 a	1.34 a	1.26	1.38 *	0.1	0.032	0.026	0.243
DL, cm	13.7 b	14.2 a *	13.7 b	14.0	13.7 *	0.1	0.001	0.001	0.035
SL, cm	8.7 c*	9.1 a	8.9 b *	9.0	8.8 *	0.1	0.001	0.001	0.977

a–c—mean values of traits in rows, marked with different letters, differ significantly between lines (*p* < 0.05). *—statistically significant differences determined between males and females. TN—trunk with neck length, TL—trunk length, CC—chest circumference, KL—keel length, TB—thickness of breast muscles, DL—drumstick length, SL—shank length.

**Table 5:** Feed intake and feed conversion ratio (FCR) of Pekin ducks from conservation flocks during rearing.

Time	Genotype			Sex		SEM
	Polish Pekin P33 (*n* = 2)	Danish Pekin P8 (*n* = 2)	French Pekin P9 (*n* = 2)	Male (*n* = 3)	Female (*n* = 3)	
Feed intake (g/day)						
1–7 d	26.6	26.8	26.7	26.4	27.0	0.2
8–14 d	66.5	67.6	68.5	66.6	68.4	0.8
15–21 d	121.1	124.2	117.9	120.1	121.9	1.3
22–28 d	139.3	165.4	155.3	152.8	153.7	5.8
29–35 d	151.0	170.2	163.1	163.6	159.2	4.5
36−42 d	167.6	189.3	174.8	176.2	178.2	4.7
42–49 d	190.6	205.2	185.8	197.1	190.6	6.5
1–49 d	123.2	136.3	127.5	129.3	128.7	3.0
Feed conversion ratio (g/g)						
1–7 d	1.34	1.46	1.32	1.41	1.33	0.1
8–14 d	2.07	2.04	2.19	2.06	2.14	0.1
15–21 d	2.48	2.46	2.62	2.57	2.46	0.1
22–28 d	2.38	2.24	2.46	2.35	2.36	0.3
29–35 d	3.46	4.63	4.20	4.17	4.03	0.1
36–42 d	3.55	3.86	3.82	3.70	3.78	0.2
43–49 d	8.48	6.85	6.55	6.22	8.40	0.8
1–49 d	3.14	3.21	3.25	3.18	3.21	0.2

**Table 6:** Preslaughter body weight, carcass yield, and share (%) of carcass components of 49-d-old Pekin ducks from conservation flocks.

Feature	Genotype			Sex		SEM	*p* Value		
	Polish Pekin P33 (*n* = 14)	Danish Pekin P8 (*n* = 14)	French Pekin P9 (*n* = 14)	Male (*n* = 21)	Female (*n* = 21)		Genotype (G)	Sex (S)	G × S
Body weight, g	2007 b	2153 a	1979 b	2055	2037	24.6	0.001	0.604	0.001
Carcass weight, g	1182 b	1268 a	1227 b *	1258	1193 *	19.9	0.018	0.012	0.008
Carcass yield, %	58.9	58.9	62.0	61.2	58.6	0.8	0.086	0.067	0.626
Breast meat, %	9.6 a	9.7 a	9.0 b	9.4	9.4	0.4	0.034	0.956	0.178
Leg meat, %	12.0	12.1	12.0	12.5	11.5	0.4	0.475	0.095	0.675
Skin with fat, %	28.0	27.7	29.2	27.3	29.2	0.5	0.366	0.624	0.754
Abdominal fat, %	1.4	1.6	1.6	1.4	1.6	0.1	0.845	0.766	0.397
Neck, %	8.6 a *	8.3 b *	8.2 b *	8.8	7.9 *	0.2	0.007	0.001	0.578
Wings, %	13.2	13.5	13.9	13.8	13.3	0.2	0.672	0.056	0.950
Remainders, %	27.2 a	27.1 a	26.1 b	26.8	27.1	0.5	0.016	0.802	0.521

a, b—mean values of traits in rows, marked with different letters, differ significantly between lines (*p* < 0.05). *—statistically significant differences determined between males and females.

**Table 7:** Length and diameter of intestine segments in 49-d-old Pekin ducks from conservation flocks.

Segment	Genotype			Sex		SEM	*p* Value		
	Polish Pekin P33 (*n* = 14)	Danish Pekin P8 (*n* = 14)	French Pekin P9 (*n* = 14)	Male (*n* = 21)	Female (*n* = 21)		Genotype (G)	Sex (S)	G × S
Length, cm									
Duodenum	36.7	34.6	34.7	36.5	34.2	1.5	0.558	0.199	0.636
Jejunum	71.9	75.1	73.5	73.1	73.9	2.0	0.580	0.770	0.804
Ileum	74.5	72.9	74.5	73.6	74.3	1.6	0.806	0.751	0.814
Caeca	33.0 a	34.2 a	30.8 b	34.5	30.9 *	1.1	0.043	0.002	0.865
Colon	11.9	11.5	11.8	11.7	11.8	0.6	0.912	0.867	0.804
Total intestine	228.0	228.3	225.3	229.4	225.1	4.2	0.874	0.404	0.806
Diameter, mm									
Duodenum	6.6	6.4	6.2	6.4	6.4	0.3	0.223	0.787	0.971
Jejunum	6.1	6.4	6.3	6.1	6.4	0.2	0.535	0.322	0.664
Ileum	6.5	6.5	6.4	6.3	6.6	0.2	0.756	0.089	0.292
Caeca	4.8	4.6	4.8	5.2	4.3	0.4	0.945	0.068	0.066
Colon	7.9	8.1	7.9	8.3	7.6	0.8	0.977	0.450	0.154

a, b—mean values of traits in rows, marked with different letters, differ significantly between lines (*p* < 0.05). *—statistically significant differences determined between males and females had significantly longer caeca compared to P9 ducks. Our results also indicate that the males and females did not differ in the length of intestine and its segments except for the length of caeca.

**Table 8:** Weight and proportion (%) in the body weight of main internal organ of 49-d-old Pekin ducks from conservation flocks.

Organ	Genotype			Sex		SEM	*p* Value		
	Polish Pekin P33 (*n* = 14)	Danish Pekin P8 (*n* = 14)	French Pekin P9 (*n* = 14)	Male (*n* = 21)	Female (*n* = 21)		Genotype (G)	Sex (S)	G × S
Weight of (g)									
Gizzard	70.0	75.6	66.0	73.9	67.2	3.6	0.079	0.152	0.072
Liver	40.7 b	42.9 b	47.5 a	45.5	41.9 *	3.3	0.024	0.018	0.283
Heart	11.0	12.4	11.4	11.0	12.2	0.7	0.230	0.090	0.264
Spleen	1.0	1.1	1.0	1.1	1.0	0.1	0.119	0.765	0.783
Proportion in body weight (%)									
Gizzard	3.5 a	3.5 a	3.3 b	3.6	3.3	0.1	0.011	0.096	0.518
Liver	2.0	2.0	2.4	2.2	2.0 *	0.1	0.549	0.011	0.186
Heart	0.5	0.6	0.6	0.5	0.6	0.1	0.730	0.077	0.369
Spleen	0.05	0.05	0.05	0.05	0.06	0.1	0.763	0.425	0.997

a, b—mean values of traits in rows, marked with different letters, differ significantly between lines (*p* < 0.05). *—statistically significant differences determined between males and females.

**Table 9:** Coefficients of correlation between body weight, carcass weight, dressing percentage, carcass components and body measurements of 49-d-old Pekin ducks from conservation flocks. *p*-values are given in brackets.

Correlated Traits	Pearson’s Coefficients of Correlation, *n* = 42						
	TN, cm	TL, cm	CC, cm	KL, cm	TB, cm	DL, cm	SL, cm
BW, g	0.39 (0.001)	0.24 (0.125)	0.57 (<0.001)	0.52 (0.001)	0.22 (0.167)	0.48 (0.001)	0.28 (0.067)
CW, g	0.13 (0.402)	−0.09 (0.576)	0.26 (0.090)	0.44 (0.003)	0.33 (0.033)	0.16 (0.317)	0.07 (0.669)
DP, %	0.05 (0.742)	0.08 (0.596)	0.32 (0.036)	0.18 (0.255)	0.03 (0.842)	0.15 (0.335)	0.16 (0.314)
BM, %	0.05 (0.742)	0.08 (0.596)	0.32 (0.336)	0.18 (0.255)	0.03 (0.842)	0.35 (0.335)	0.16 (0.314)
LM, %	0.18 (0.244)	0.18 (0.246)	0.29 (0.060)	0.13 (0.423)	−0.08 (0.594)	0.30 (0.054)	0.18 (0.259)
SF, %	−0.01 (0.937)	0.16 (0.321)	−0.01 (0.930)	−0.09 (0.557)	0.04 (0.786)	−0.02 (0.920)	−0.06 (0.694)
AF, %	−0.12 (0.443)	−0.11 (0.495)	0.19 (0.224)	0.15 (0.326)	0.07 (0.650)	0.11 (0.484)	0.15 (0.334)
NP, %	0.21 (0.179)	0.30 (0.055)	0.04 (0.790)	−0.20 (0.200)	−0.30 (0.051)	0.23 (0.143)	0.19 (0.219)
WP, %	0.21 (0.172)	0.22 (0.163)	0.19 (0.219)	0.06 (0.707)	−0.08 (0.619)	0.13 (0.399)	0.19 (0.229)
RP, %	−0.19 (0.209)	−0.43 (0.005)	−0.14 (0.374)	0.28 (0.070)	0.29 (0.061)	−0.19 (0.227)	−0.12 (0.439)

BW—body weight, CW—carcass weight, DP—dressing percentage, BM—breast muscle percentage, LM—leg muscle percentage, SF—percentage of skin with subcutaneous fat, AF—percentage of abdominal fat, NP—neck percentage, WP—wing percentage, RP—remainders percentage, TN—trunk with neck length, TL—trunk length, CC—chest circumference, KL—keel length, TB—thickness of breast muscles, DL—drumstick length, SL—shank length.

**Table 10:** Coefficients of correlation between intestinal measurements and body weight and dimensions of 49-d-old Pekin ducks from conservation flocks, *p*-values are given in brackets.

Correlated Traits	Pearson’s Coefficients of Correlation, *n* = 42							
	BW, g	TN, cm	TL, cm	CC, cm	KL, cm	TB, cm	DL, cm	SL, cm
DL, cm	−0.17 (0.263)	0.08 (0.617)	−0.19 (0.226)	−0.30 (0.053)	−0.03 (0.856)	−0.27 (0.080)	−0.15 (0.360)	−0.19 (0.238)
IL, cm	0.23 (0.139)	0.17 (0.265)	−0.03 (0.833)	0.18 (0.244)	0.19 (0.222)	−0.09 (0.537)	0.21 (0.187)	0.29 (0.063)
JL, cm	−0.15 (0.329)	0.02 (0.892)	−0.13 (0.428)	0.04 (0.800)	−0.18 (0.243)	0.02 (0.873)	−0.05 (0.772)	0.02 (0.898)
CL, cm	0.04 (0.735)	0.54 (0.001)	0.18 (0.247)	0.12 (0.454)	0.07 (0.650)	−0.24 (0.131)	0.16 (0.325)	0.43 (0.004)
KL, cm	−0.07 (0.626)	0.06 (0.674)	0.04 (0.778)	−0.01 (0.937)	0.02 (0.911)	0.01 (0.985)	−0.19 (0.224)	−0.12 (0.456)
TL, cm	−0.07 (0.641)	0.30 (0.051)	−0.03 (0.834)	−0.03 (0.851)	−0.06 (0.702)	−0.24 (0.136)	−0.07 (0.649)	0.13 (0.397)
DD, mm	0.01 (0.987)	0.01 (0.944)	0.29 (0.063)	0.21 (0.180)	0.13 (0.426)	0.02 (0.881)	0.04 (0.816)	0.13 (0.397)
ID, mm	0.08 (0.583)	−0.15 (0.329)	0.01 (0.938)	0.08 (0.606)	0.09 (0.561)	0.04 (0.799)	−0.01 (0.969)	0.21 (0.174)
JD, mm	0.23 (0.131)	−0.15 (0.332)	0.06 (0.706)	0.22 (0.165)	0.34 (0.025)	0.29 (0.057)	0.15 (0.345)	0.09 (0.567)
CD, mm	0.04 (0.789)	0.11 (0.471)	0.15 (0.344)	0.20 (0.197)	0.29 (0.063)	0.19 (0.212)	0.08 (0.610)	0.52 (0.001)
KD, mm	−0.08 (0.598)	0.07 (0.625)	0.08 (0.621)	−0.15 (0.353)	0.16 (0.300)	0.07 (0.631)	−0.20 (0.199)	0.14 (0.384)

DL—duodenum length, IL—ileum length, JL—jejunal length, CL—caeca length, KL—colon length, TL— total intestinal length, DD—duodenum diameter, ID—ileum diameter, JD—jejunal diameter, CD—caeca diameter, KD—colon diameter, BW—body weight, TN—trunk with neck length, TL—trunk length, CC—chest circumference, KL—keel length, TB—thickness of breast muscles, DL—drumstick length, SL—shank length.

**Table 11:** Coefficients of correlation between percentage of some internal organs (g, %) and body weight and measurements of 49-d-old Pekin ducks from conservation flocks. *p*-values are given in brackets.

Correlated Traits	Pearson’s Coefficients of Correlation, *n* = 42							
	BW, g	TN, cm	TL, cm	CC, cm	KL, cm	TB, cm	DL, cm	SL, cm
GW, cm	−0.18 (0.249)	−0.19 (0.216)	−0.11 (0.489)	−0.09 (0.544)	0.14 (0.385)	0.01 (0.994)	−0.38 (0.011)	−0.13 (0.404)
LW, cm	0.15 (0.351)	0.08 (0.582)	0.18 (0.252)	0.02 (0.895)	0.10 (0.522)	0.27 (0.086)	−0.14 (0.380)	0.02 (0.890)
HW, cm	0.20 (0.208)	0.38 (0.012)	0.24 (0.126)	−0.04 (0.780)	0.22 (0.165)	0.11 (0.502)	−0.29 (0.064)	−0.22 (0.160)
SW, cm	0.18 (0.241)	0.05 (0.756)	−0.01 (0.988)	0.16 (0.309)	0.13 (0.408)	0.01 (0.989)	0.19 (0.237)	0.20 (0.209)
GP, cm	−0.31 (0.049)	−0.24 (0.132)	−0.23 (0.149)	−0.08 (0.633)	0.06 (0.715)	−0.09 (0.531)	−0.35 (0.025)	−0.09 (0.558)
LP, cm	0.08 (0.607)	0.07 (0.670)	0.13 (0.402)	0.03 (0.834)	0.04 (0.795)	0.21 (0.178)	−0.10 (0.547)	0.05 (0.730)
HP, mm	−0.03 (0.837)	−0.08 (0.583)	−0.10 (0.509)	0.24 (0.133)	−0.02 (0.892)	−0.12 (0.459)	0.18 (0.255)	0.06 (0.698)
SP, mm	0.01 (0.975)	−0.15 (0.338)	−0.02 (0.884)	0.06 (0.718)	−0.02 (0.918)	0.13 (0.403)	−0.028 (0.865)	0.06 (0.698)

BW—body weight, TN—trunk with neck length, TL—trunk length, CC—chest circumference, KL—keel length, TB—thickness of breast muscles, DL—drumstick length, SL—shank length, GW—gizzard weight, LW—liver weight, HW—heart weight, SW—spleen weight, GP—proportion of gizzard, LP—proportion of liver, HP—proportion of heart, SP—proportion of spleen.

**Table 12:** Coefficients of correlation between intestinal measurements and carcass traits of 49-d-old Pekin ducks from conservation flocks, *p*-values are given in brackets.

Correlated Traits	Pearson’s Coefficients of Correlation, *n* = 42								
	CW, g	DP, %	BM, %	LM, %	SF, %	AF, %	NP, %	WP, %	RP, %
DL, cm	−0.21 (0.181)	−0.11 (0.480)	−0.12 (0.449)	0.03 (0.839)	−0.06 (0.683)	−0.23 (0.135)	0.19 (0.219)	−0.11 (0.478)	0.16 (0.302)
IL, cm	0.30 (0.052)	0.16 (0.309)	−0.14 (0.357)	0.04 (0.804)	−0.12 (0.450)	0.13 (0.414)	−0.36 (0.021)	−0.18 (0.245)	0.04 (0.805)
JL, cm	−0.03 (0.864)	0.14 (0.393)	0.03 (0.836)	0.14 (0.365)	0.04 (0.822)	−0.02 (0.874)	−0.07 (0.667)	−0.23 (0.143)	0.12 (0.433)
CL, cm	−0.01 (0.970)	−0.10 (0.546)	0.09 (0.562)	0.12 (0.430)	−0.10 (0.546)	0.01 (0.991)	0.12 (0.431)	0.06 (0.694)	−0.06 (0.721)
KL, cm	0.08 (0.602)	0.14 (0.389)	−0.03 (0.855)	0.28 (0.072)	−0.33 (0.032)	−0.22 (0.165)	0.02 (0.888)	−0.12 (0.453)	−0.09 (0.583)
TL, cm	−0.09 (0.583)	0.02 (0.880)	−0.01 (0.954)	−0.01 (0.958)	−0.07 (0.659)	−0.17 (0.279)	0.01 (0.967)	−0.25 (0.117)	0.10 (0.524)
DD, mm	−0.16 (0.325)	−0.22 (0.171)	0.19 (0.223)	0.09 (0.552)	0.03 (0.835)	0.01 (0.989)	0.12 (0.481)	0.10 (0.525)	−0.17 (0.271)
ID, mm	0.10 (0.524)	0.09 (0.578)	−0.05 (0.735)	−0.05 (0.743)	−0.03 (0.854)	−0.01 (0.937)	−0.20 (0.206)	−0.09 (0.582)	0.06 (0.692)
JD, mm	0.06 (0.700)	−0.12 (0.435)	0.05 (0.767)	0.04 (0.789)	0.05 (0.735)	0.13 (0.429)	−0.08 (0.606)	0.10 (0.517)	0.13 (0.424)
CD, mm	−0.08 (0.605)	−0.08 (0.591)	−0.07 (0.674)	0.13 (0.400)	−0.07 (0.659)	0.03 (0.842)	0.08 (0.585)	0.20 (0.200)	0.13 (0.393)
KD, mm	0.01 (0.944)	−0.05 (0.752)	0.13 (0.416)	−0.06 (0.695)	0.08 (0.633)	0.21 (0.182)	−0.10 (0.506)	0.02 (0.899)	0.11 (0.505)

CW—carcass weight, DP—dressing percentage, BM—breast muscle percentage, LM—leg muscle percentage, SF—percentage of skin with subcutaneous fat, AF—percentage of abdominal fat, NP—neck percentage, WP—wing percentage, RP—remainders percentage, DL—duodenum length, IL—ileum length, JL—jejunal length, CL—caeca length, KL—colon length, TL—total intestinal length, DD—duodenum diameter, ID—ileum diameter, JD—jejunum diameter, CD—caeca diameter, KD—colon diameter.

**Table 13:** Coefficients of correlation between percentage of some internal organs (g, %) and carcass traits of 49-d-old Pekin ducks from conservation flocks. *p*-values are given in brackets.

Correlated Traits	Pearson’s Coefficients of Correlation, *n* = 42								
	CW g	DP %	BM %	LM %	SF %	AF %	NP %	WP %	RP %
GW, g	−0.23 (0.144)	−0.21 (0.183)	0.24 (0.131)	−0.11 (0.506)	0.14 (0.370)	0.09 (0.558)	−0.03 (0.852)	0.01 (0.999)	0.01 (0.999)
LW, g	0.02 (0.869)	−0.11 (0.497)	−0.13 (0.414)	−0.24 (0.118)	0.14 (0.360)	−0.08 (0.614)	−0.01 (0.928)	0.07 (0.632)	−0.05 (0.769)
HW, g	0.17 (0.282)	0.01 (0.944)	0.16 (0.324)	0.16 (0.324)	0.18 (0.246)	−0.15 (0.329)	−0.01 (0.969)	−0.03 (0.836)	0.01 (0.999)
SW, g	0.07 (0.633)	−0.10 (0.517)	0.46 (0.002)	0.14 (0.387)	0.06 (0.713)	−0.11 (0.492)	0.15 (0.345)	0.27 (0.085)	−0.13 (0.412)
GP, %	−0.28 (0.079)	−0.17 (0.279)	0.12 (0.428)	−0.11 (0.457)	0.09 (0.547)	0.13 (0.393)	−0.08 (0.619)	0.05 (0.768)	0.18 (0.244)
LP, %	0.01 (0.959)	−0.07 (0.646)	−0.21 (0.183)	−0.21 (0.183)	0.10 (0.517)	−0.06 (0.682)	−0.04 (0.815)	0.10 (0.518)	−0.06 (0.700)
HP, %	0.09 (0.589)	0.14 (0.382)	0.30 (0.056)	0.16 (0.314)	−0.02 (0.906)	−0.16 (0.305)	0.07 (0.676)	0.01 (0.924)	−0.15 (0.329)
SP, %	−0.01 (0.988)	−0.08 (0.636)	0.44 (0.004)	−0.03 (0.886)	0.09 (0.567)	−0.09 (0.544)	0.07 (0.637)	0.19 (0.234)	−0.17 (0.289)

CW—carcass weight, DP—dressing percentage, BM—breast muscle percentage, LM—leg muscle percentage, SF—percentage of skin with subcutaneous fat, AF—percentage of abdominal fat, NP—neck percentage, WP—wing percentage, RP—remainders percentage, GW—gizzard weight, LW—liver weight, HW—heart weight, SW—spleen weight, GP—proportion of gizzard, LP—proportion of liver, HP—proportion of heart, SP—proportion of spleen.
